# DOT1L Inhibition Sensitizes MLL-Rearranged AML to Chemotherapy

**DOI:** 10.1371/journal.pone.0098270

**Published:** 2014-05-23

**Authors:** Wei Liu, Lisheng Deng, Yongcheng Song, Michele Redell

**Affiliations:** 1 Department of Pediatrics, Section of Hematology-Oncology, Baylor College of Medicine, Houston, Texas, United States of America; 2 Department of Pharmacology, Baylor College of Medicine, Houston, Texas, United States of America; Queen's University Belfast, United Kingdom

## Abstract

DOT1L, the only known histone H3-lysine 79 (H3K79) methyltransferase, has been shown to be essential for the survival and proliferation of *mixed-linkage leukemia* (*MLL*) gene rearranged leukemia cells, which are often resistant to conventional chemotherapeutic agents. To study the functions of DOT1L in MLL-rearranged leukemia, SYC-522, a potent inhibitor of DOT1L developed in our laboratory, was used to treat MLL-rearranged leukemia cell lines and patient samples. SYC-522 significantly inhibited methylation at H3K79, but not H3K4 or H3K27, and decreased the expression of two important leukemia-relevant genes, *HOXA9* and *MEIS1*, by more than 50%. It also significantly reduced the expression of *CCND1* and *BCL2L1*, which are important regulators of cell cycle and anti-apoptotic signaling pathways. Exposure of MLL-rearranged leukemia cells to this compound caused cell cycle arrest and promoted differentiation of those cells, both morphologically and by increased CD14 expression. SYC-522 did not induce apoptosis, even at 10 µM for as long as 6 days. However, treatment with this DOT1L inhibitor decreased the colony formation ability of primary MLL-rearranged AML cells by up to 50%, and promoted monocytic differentiation. Notably, SYC-522 treatment significantly increased the sensitivity of MLL-rearranged leukemia cells to chemotherapeutics, such as mitoxantrone, etoposide and cytarabine. A similar sensitization was seen with primary MLL-rearranged AML cells. SYC-522 did not affect chemotherapy-induced apoptosis in leukemia cells without MLL-rearrangement. Suppression of DOT1L activity inhibited the mitoxantrone-induced increase in the DNA damage response marker, γH2AX, and increased the level of cPARP, an intracellular marker of apoptosis. These results demonstrated that SYC-522 selectively inhibited DOT1L, and thereby altered gene expression, promoted differentiation, and increased chemosensitivity by preventing DNA damage response. Therefore, inhibition of DOT1L, in combination with DNA damaging chemotherapy, represents a promising approach to improving outcomes for MLL-rearranged leukemia.

## Introduction

Acute leukemia with somatic rearrangements of the gene *Mixed-lineage leukemia (MLL)* involves a chromosomal translocation that fuses the MLL gene at 11q23 with one of >70 reported fusion partners [1]. Normally, MLL plays a positive role in maintenance of Hox gene expression during development [2]. For MLL fusion proteins, the 5′ end of the MLL gene is fused to the 3′ portion of its partners, such as AF4, AF9, AF10 or ENL [3]. MLL-rearranged leukemia accounts for ∼75% of infant and ∼10% child/adult acute leukemias [4]. This type of leukemia has a particularly poor prognosis and high risk of relapse. The 5-year event-free survival rates of infants with MLL-rearranged acute lymphoid leukemia (ALL) are only 30–40% [5], and the 5-year-event-free survival rates for patients with MLL-rearranged acute myeloid leukemia (AML) are 34–61% [6]. Intensified chemotherapies have led to increased toxicity without significantly improved survival. There is thus a pressing need to find new drugs to treat patients with MLL-rearranged leukemia.

DOT1L, the only known histone 3 lysine 79 (H3K79) methyltransferase, has been reported to interact with MLL-AF10 [7], and is required for initiation and maintenance of several types of MLL-rearranged leukemias, such as MLL-AF9 and MLL-AF6 [8], [9], [10]. DOT1L activity in MLL-rearranged leukemia leads to H3K79 hypermethylation, resulting in aberrant expression of genes related to hematopoietic cell stemness and self-renewal [11], [12]. Thus, the aberrant gene expression caused by H3K79 methylation contributes to dysregulated hematopoietic differentiation and leukemogenesis. Moreover, methylation of H3K79 by DOT1L has been shown to facilitate DNA damage repair by altering the chromatin structure and/or by recruiting proteins that mediate repair of DNA double strand breaks (DSBs) [13], [14]. Effective DNA damage signaling has been associated with chemoresistance in several cancers [15], [16]. Therefore, we proposed that inhibition of DOT1L activity may sensitize MLL-rearranged cells to chemotherapy via suppressing DNA damage repair.

Since DOT1L methyltransferase activity is critical to MLL-rearranged leukemia [7], inhibition of DOT1L may provide a potential therapy for this type of leukemia. Indeed, several other DOT1L inhibitors have been reported to induce apoptosis of MLL-rearranged leukemia cell lines [17], [18]. Our medicinal chemistry studies identified a small-molecule compound, SYC-522, that is a potent and selective inhibitor of DOT1L with a *Ki* value of 0.5 nM (compound 55 in our prior publication [19]). Here, we report the biological activities of SYC-522 in several MLL-rearranged leukemia cell lines and human leukemia primary samples. Similar to the Epizyme compounds [17], [18], SYC-522 decreased *HOXA9* and *MEIS1* gene expression and promoted cell differentiation. In contrast to the Epizyme compounds, SYC-522 did not induce significant apoptosis, but instead sensitized the cells to chemotherapeutic drugs by inhibiting the DNA damage response.

## Materials and Methods

### Ethics Statement

Primary MLL-rearranged AML and MLL-rearranged ALL cells were acquired from the Research Tissue Support Service at Texas Children's Hospital. Samples came from patients treated at Texas Children's Hospital, whose families gave informed consent, in accordance with the Declaration of Helsinki, for remainder marrow to be used for research. Normal bone marrow (NBM) was obtained from healthy individuals donating marrow for patients at Texas Children's Hospital. In all cases, mononuclear cells were enriched by density centrifugation and cryopreserved. Studies with human samples were approved by the Institutional Review Board of Baylor College of Medicine.

### Cell Culture

Human MLL-rearranged acute myeloid leukemia cell lines MV4-11 and MOLM13, as well as NB4 and HL-60 without MLL rearrangement, were maintained in a humidified incubator with 5% CO_2_, at 37°C. MOLM13, NB4 and HL-60 cells were grown in high-glucose RPMI 1640 (ATCC) with 10% FBS. MV4-11 cells were grown in standard RPMI 1640 (Invitrogen) with 10% FBS and 0.45% sucrose. All media were supplemented with 2 mM L-glutamine and penicillin/streptomycin. All the cell culture reagents were purchased from Invitrogen Life Technologies.

### Western Blotting

For assessment of the effect of SYC-522 on histone methylation in MLL-rearranged leukemia cell lines, 1×10^6^ cells were incubated with SYC-522 for 3–6 days. The doses for treating the cells were determined by IC_50_s reported previously [19]. MV4-11 cells were treated with 3 µM SYC-522, and MOLM13 were treated with 10 µM. Cells were harvested at the appropriate time point and histones were extracted as described [18]. The concentrations of extracted histones were measured by Bradford protein assay. The dimethylation of H3K79 (Abcam 3594), trimethylation of H3K4 (Cell Signaling Technology 9751), trimethylation of H3K27 (Cell Signaling Technology 9733) and total H3 (Cell Signaling Technology 3638) were probed by appropriate primary antibodies (1∶1000 dilution), followed by IR700 or IR800 goat anti-rabbit IgG or goat anti-mouse IgG secondary antibodies (LI-COR). Bands were visualized and quantified on the Odyssey Infrared Imager. Densitometry values for methylated H3K4, H3K27 and H3K79 were normalized to the corresponding total H3 value, and shown as the percentage of the value for untreated cells.

### Quantitative Real-Time PCR

MLL-rearranged cells MV4-11 and MOLM13 were treated with SYC-522 at appropriate doses. Total RNA was extracted with Rneasy Plus Micro kit (Qiagen), then reverse-transcribed with Taqman Reverse-Transcript Reagents kit (Life Technologies). In some cases, mRNA extraction and cDNA synthesis were done with μMACS One-step cDNA kit (Miltenyi Biotec). Taqman primer/probe sets for genes of interest were purchased from Applied Biosystems (HOXA9 Hs00266821_m1, MEIS1 Hs01017441_m1, BCL2L1 Mm00437783_m1, CCND1 Hs00765553_m1, 18S 4319413E-0909045). Samples were run on an ABI7900 HT Fast Real Time PCR machine (Baylor College of Medicine Molecular Core Lab). Target gene cycle thresholds (Ct) were normalized by subtracting the 18S Ct to get ΔCt value. The ΔCt values were calibrated by subtracting untreated ΔCt from SYC-522-treated ΔCt to get ΔΔCt. The fold change was calculated as 2^(−ΔΔCt)^.

### Cell Cycle Analysis

MV4-11 cells were plated at 5×10^4^ cells/ml in a 24-well plate. Cells were incubated with 3 µM SYC-522 for up to 9 days. On day 6, two thirds of cells and medium were removed from the 24-well plate and replaced with fresh drug in fresh medium. Cells were washed with PBS and fixed with 70% ethanol at 4°C overnight. Then the cells were stained with propidium iodide and 625 µg/ml Rnase A for 1 h. The stained cells were analyzed by flow cytometry (FACScan, BD) and FCS Express 4 software (DeNovo).

### Cell Apoptosis Analysis

MV4-11, MOLM13, NB4 and HL-60 cells were plated at 5×10^4^ cells/ml in a 24-well plate. Cells were incubated with SYC-522 at appropriate doses (3 µM for MV4-11, 10 µM for MOLM13, NB4 and HL-60) for up to 6 days. On day 3 or 6, mitoxantrone (10 nM or 100 nM), etoposide (100 nM or 1 µM) or cytarabine (3 µM or 30 µM) was added to the cells. 24 h after this combination treatment with chemotherapy drugs and SYC-522, cells were harvested and labeled with Annexin V-FITC (BD), and the percent of apoptotic cells measured by flow cytometry (FACScan). The apoptosis rate in untreated cells was subtracted from that of drug-treated cells to determine the percent of apoptosis attributable to drug treatment.

### Analysis of CD14 expression

MV4-11 and MOLM13 cells were plated in 24-well plates, and media and SYC-522 were replaced every 6 days as described above. The cells were harvested every 3 days until 18 days to measure the expression of CD14 in the cells. The cells were washed with flow buffer (PBS with 0.2% BSA and 0.09% sodium azide) and labeled with CD14-PE antibody (BD 562691) for 45 min at 4°C. Data were acquired on the FACScan and analyzed with FCS Express 4 software.

### Clonogenic Assay

Primary MLL-rearranged AML cells were plated in Methocult H4434 (Stem Cell Technologies) methylcellulose medium at 10^4^ per ml in duplicate 35 mm dishes. Primary MLL-rearranged ALL cells were plated in Methocult H4325 (Stem Cell Technologies) with IL-7 (20 ng/ml, Stem Cell Technologies), stem cell factor (30 ng/ml, Stem Cell Technologies) and Flt ligand (20 ng/ml, Cell Signaling Technology) at 10^4^ per ml in duplicate dishes. Normal bone marrow cells were plated in Methocult H4434 at 1×10^6^ per ml in duplicate dishes. SYC-522 was added directly to the methylcellulose medium, in increasing concentrations (0, 30 nM, 300 nM, 3 µM, 30 µM), or DMSO as a control. For chemotherapy +SYC-522 combination experiments, chemotherapy drugs (etoposide 100 nM or 1 µM, mitoxantrone 10 nM or 100 nM) and/or SYC-522 (10 µM) were added directly to the methylcellulose. The colonies were counted on day 14. MLL-rearranged AML cells were harvested and cytospun onto glass slides, then stained with Wright Giemsa on day 14.

### DNA damage response assay

MV4-11 and MOLM13 were incubated with SYC-522 at appropriate doses (3 µM for MV4-11, 10 µM for MOLM13) for up to 6 days. On day 3 or 6, 100 nM mitoxantrone was added to the cells for 4 h. Then the drugs were washed away with PBS, and cells were incubated in fresh medium for another 12 h. The cells were fixed in 2% paraformaldehyde and permeabilized in ice cold 100% methanol, as described [20]. Cells were labeled with γH2AX- AlexaFluor488 (BD) and cPARP− AlexaFluor647 (BD). Data were acquired on the LSRII (BD) and analyzed with FCS Express4.

### Statistics

Results from treated v. control groups were compared by independent Student's t-test. All values shown in figures are mean ±SEM. For the clonogenic assay, one-way ANOVA followed by Tukey's post tests were performed using GraphPad Prism 5. Comparisons with p<0.05 were considered to be significantly different.

## Results

### SYC-522 inhibited histone methylation at H3K79, but not H3K4 or H3K27

SYC-522 was a potent and highly selective DOT1L inhibitor in an *in vitro* enzyme inhibition assay, which showed that the Ki value for DOT1L was 0.5 nM, and Ki values for three structurally similar histone methyltransferases (HMTs), PRMT1, CARM1 and SUV39H1, were more than 100 µM [19]. In this study, we have evaluated the compound in leukemia cell-based functional assays. MV4-11 and MOLM13 are MLL-rearranged AML cell lines with different fusion partners: MV4-11 cells express MLL-AF4, and MOLM13 cells express MLL-AF9. Based on our published IC_50_s for growth inhibition in MV4-11 cells (4.4 µM) compared to other MLL-rearranged cells (∼10 µM) [19], we used 3 µM SYC-522 for MV4-11 cells and 10 µM SYC-522 for MOLM-13 cells. A similar difference in potency between MV4-11 and MOLM-13 cells was reported for the Epizyme compounds [17], [18]. It is not clear why MV4-11 cells are somewhat more sensitive to these inhibitors, although it is unlikely to be due to the difference in fusion partners, since other cell lines with the MLL-AF4 fusion (e.g. RS4-11) are not as sensitive as MV4-11 cells [17], [18].

**Figure 1:**
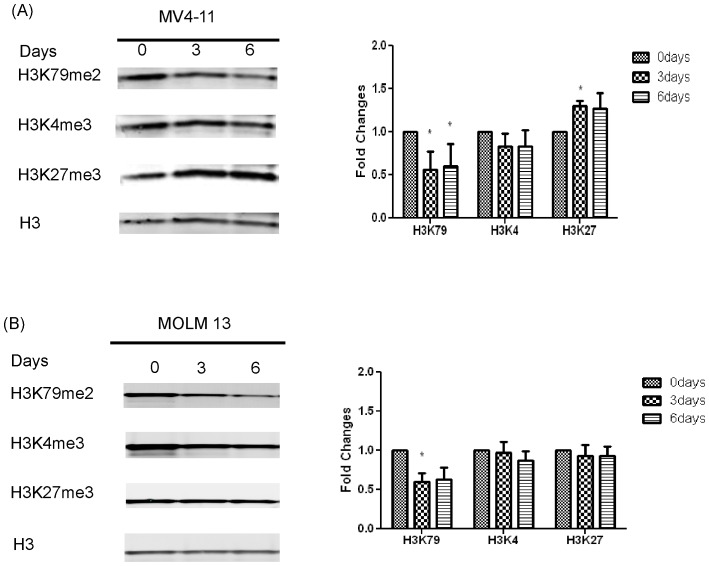
SYC-522 decreased the methylation of H3K79 in MLL-rearranged leukemia cell lines. (A) MV4-11 cells were treated with 3 µM SYC-522 and (B) MOLM13 cells were treated with 10 µM SYC-522. The histone proteins were extracted from the cell lysates after 0, 3, or 6 days of treatment. H3K79 di-methylation, H3K4 tri-methylation and H3K27 tri-methylation were assessed using specific antibodies. Representative Western blots are shown on the left, and averaged densitometry values are shown on the right. Levels of methylated H3K4, H3K27 and H3K79 were normalized to the corresponding total H3 and shown as a fold change of values for untreated cells. Bars represent the mean ±SEM of 3 independent experiments. *: p<0.05, day 0 vs. day 3 or 6, unpaired t-test.

First, we tested the ability of SYC-522 to inhibit di-methylation of H3K79 (H3K79me2), as well as tri-methylation of H3K4 (H3K4me3) and H3K27 (H3K27me3) as a comparison, since H3K4me3 is also involved in HOX gene regulation [21] and H3K27me3 contributes to the suppression of cell differentiation [22]. After treatment for up to 6 days, H3K79me2 was reduced significantly in both cell lines (Fig. 1). In contrast, SYC-522 treatment only slightly reduced H3K4me3 in these leukemia cells (Fig. 1). Though increased H3K27me3 was observed in MV4-11cells, the change was small (Fig. 1A) and there was no significant change in MOLM13 (Fig. 1B). Together with our previous study [19], these results confirmed that SYC-522 was a selective inhibitor of H3K79 methylation.

### SYC-522 blocked the expression of genes involved in leukemogenesis, cell cycle and anti-apoptosis

**Figure 2:**
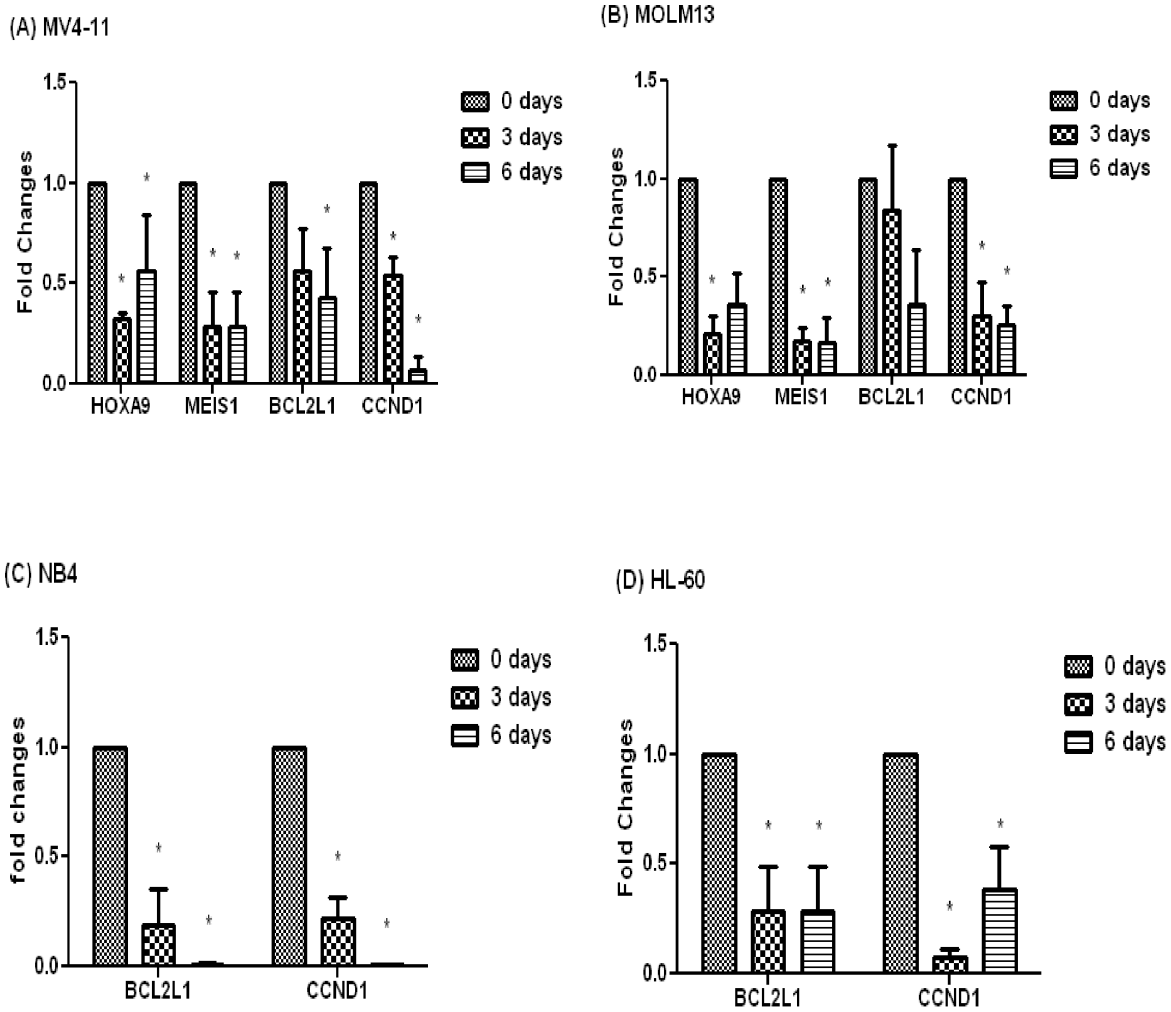
SYC-522 decreased the expression of key genes in leukemia cell lines. *HOXA9*, *MEIS1*, *CCND1* and *BCL2L1* mRNA levels in (A) MV4-11 and (B) MOLM13 cells were analyzed by quantitative real-time PCR at 0, 3, or 6 days after SYC-522 treatment. *HOXA9* and *MEIS1* expression were not detectable in (C) NB4 and (D) HL-60 cells. Only *CCND1* and *BCL2L1* mRNA expression were analyzed in these two cell lines. The mRNA expression was normalized to the level of 18S. Values represent the fold change in mRNA levels in treated vs. control cells. Bars represent the mean ±SEM of 3 independent experiments. *: p<0.05, day 0 vs. day 3 or 6, unpaired t-test.

Next, we tested whether SYC-522 inhibited the expression of MLL-fusion target genes *HOXA9* and *MEIS1*, both of which have been found to be overexpressed in MLL-rearranged leukemia [23], [24] and downregulated by treatment with a DOT1L inhibitor [18]. Quantitative real-time PCR demonstrated that the expression levels of *HOXA9* and *MEIS1* were decreased in MV4-11 and MOLM13 cells by 50% or greater after 3–6 days of treatment with SYC-522 (Fig. 2A and 2B). The expression of these two genes in NB4 and HL-60 cells without MLL rearrangement was undetectable. These results showed that inhibition of DOT1L by SYC-522 was able to reverse the overexpression of these two key leukemogenic genes.

We also investigated the expression of *CCND1* (cyclin D1) and *BCL2L1* (Bcl-xL), which are key regulators of cell cycle and anti-apoptotic signaling pathways, respectively. The expression of both was reduced in leukemia cell lines after SYC-522 treatment, regardless of the MLL status (Fig. 2). Cell cycle analysis confirmed that DOT1L inhibition by SYC-522 caused MV4-11 cells to be arrested at the G0/G1 phase after a 3-day treatment (Fig. S1), with the percentage of MV4-11 cells in the G0/G1 phase significantly increased, while the percentage in the S phase was decreased. These results suggested that SYC-522 treatment could affect the cell cycle and apoptosis signaling in both types of leukemia cells, indicating that DOT1L inhibitors may have some degree of activity against leukemia cells without MLL rearrangement.

### SYC-522 inhibited the colony formation ability of primary MLL-rearranged leukemia cells

**Figure 3:**
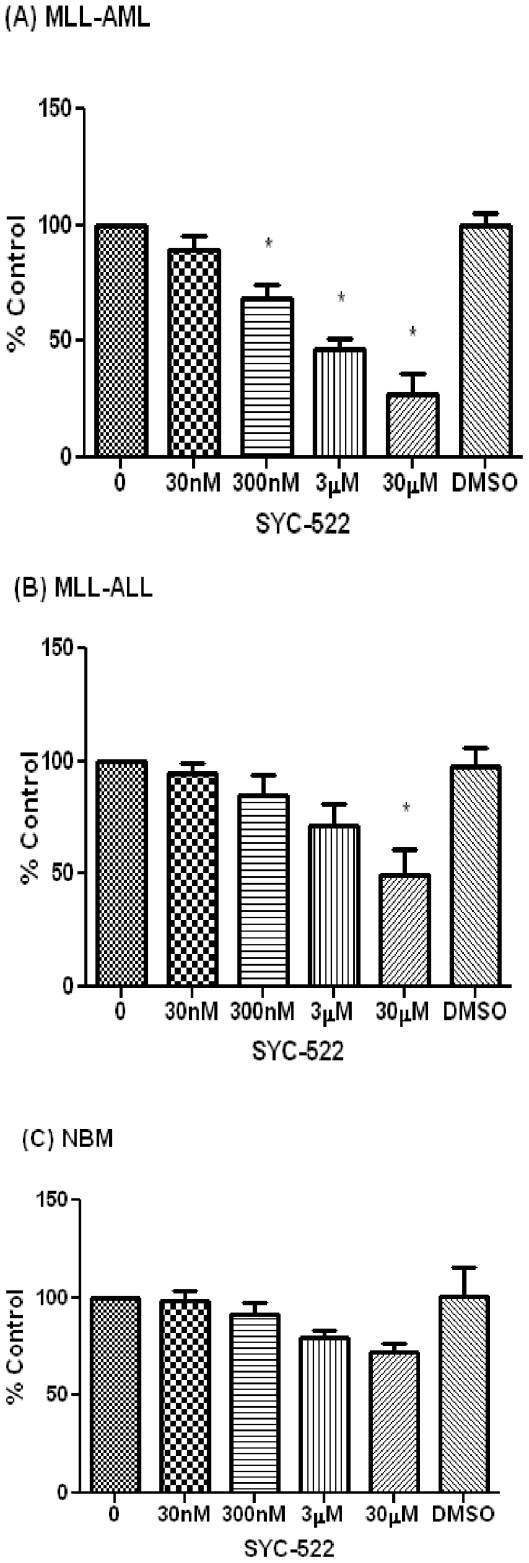
SYC-522 inhibited the colony formation ability of primary MLL-rearranged leukemia cells. Bar charts show the average percent change in CFU numbers in (A) three independent primary MLL-rearranged AML samples, (B) three independent primary MLL-rearranged ALL samples, and (C) three independent normal bone marrow (NBM) samples, after SYC-522 treatment. CFUs were counted after 14 days and the values from duplicate dishes were averaged and expressed as percent of the untreated control. Values represent the mean ±SEM of 3 independent primary samples *: p<0.05, untreated vs. SYC-522 treated, One-way ANOVA followed by Tukey's post tests.

To further determine the activity of SYC-522 against more clinically relevant cells, we tested whether it impaired the growth of leukemic cells in a colony-forming assay using primary MLL-rearranged leukemia cells. In addition, to characterize the potential toxicity of SYC-522, its activity against normal human bone marrow cells was also examined. After 14 days of culture in methylcellulose supplemented with cytokines (Methocult H4435, Stem Cell Technologies), there was a dose-dependent decrease in the number of granulocyte-monocyte colony forming units (CFU-GM) in cells treated with SYC-522 compared to the DMSO control. CFU-GM were decreased by about 10% at doses as low as 30 nM, and at the highest dose (30 µM), CFU-GM were decreased by about 70% (Fig. 3A). In contrast, even the highest doses had only a minimal effect on colony numbers in normal bone marrow samples (Fig. 3C). MLL-rearranged ALL samples also were less sensitive to SYC-522 than MLL-rearranged AML samples. At 30 µM, the colony numbers of primary MLL-rearranged ALL cells were only reduced by about 40%, and lower doses had little effect on ALL colony numbers (Fig. 3B). These results suggested that MLL-rearranged AML may be more sensitive to the inhibitory effect of SYC-522 compared to MLL-rearranged ALL, and that its toxicity to normal bone marrow cells may be minimal.

### SYC-522 promoted the differentiation of MLL-rearranged leukemia cell lines

**Figure 4:**
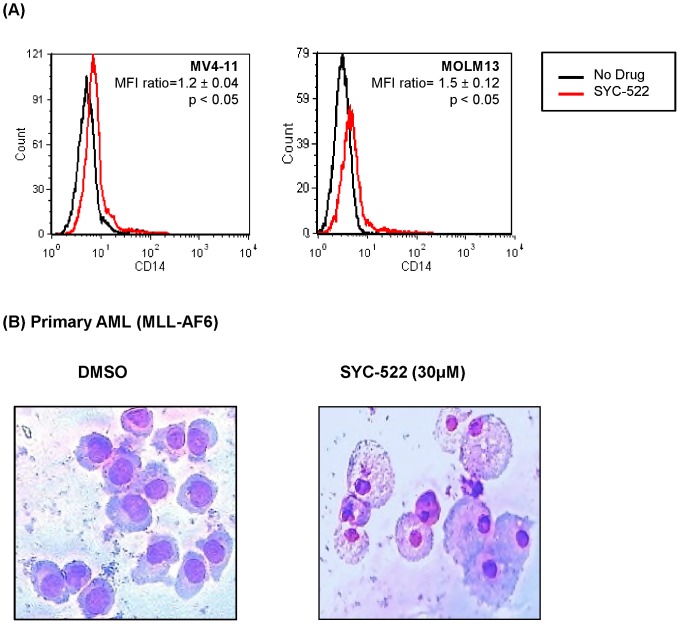
SYC-522 promoted the differentiation of MLL-rearranged leukemia cell lines and primary MLL-rearranged AML cells. (A) MV4-11 cells were treated with 3 µM SYC-522 and MOLM13 cells were treated with 10 µM SYC-522 for up to 18 days. Cells were analyzed by flow cytometry for CD14 expression. MFI (mean fluorescence intensity) ratio = MFI (SYC-522 treated)/MFI (untreated), n = 3 independent experiments, *p<0.05, unpaired t-test; (B) Primary MLL-rearranged AML cells were harvested after clonogenic assays and stained with Wright Giemsa (original magnification 40X).

MV4-11 and MOLM13 are monocytic leukemia cells. Thus, the differentiation of these cell lines can be monitored by the cell surface expression of CD14. As shown in Figure 4, the expression of CD14 was significantly increased after SYC-522 treatment in MV4-11 and MOLM13 (Fig. 4A). Furthermore, we stained primary MLL-rearranged AML cells with Wright-Giemsa after 14 days of culture in methylcellulose with and without SYC-522. There were few differentiated cells in untreated dishes, while the cells treated with 30 µM SYC-522 had small nuclei with abundant cytoplasm and vacuoles, representing differentiated monocytes (Fig. 4B). These results demonstrated that SYC-522 promoted the differentiation of MLL-rearranged leukemia cells.

### SYC-522 sensitized MLL-rearranged cells to chemotherapeutic agents

**Figure 5:**
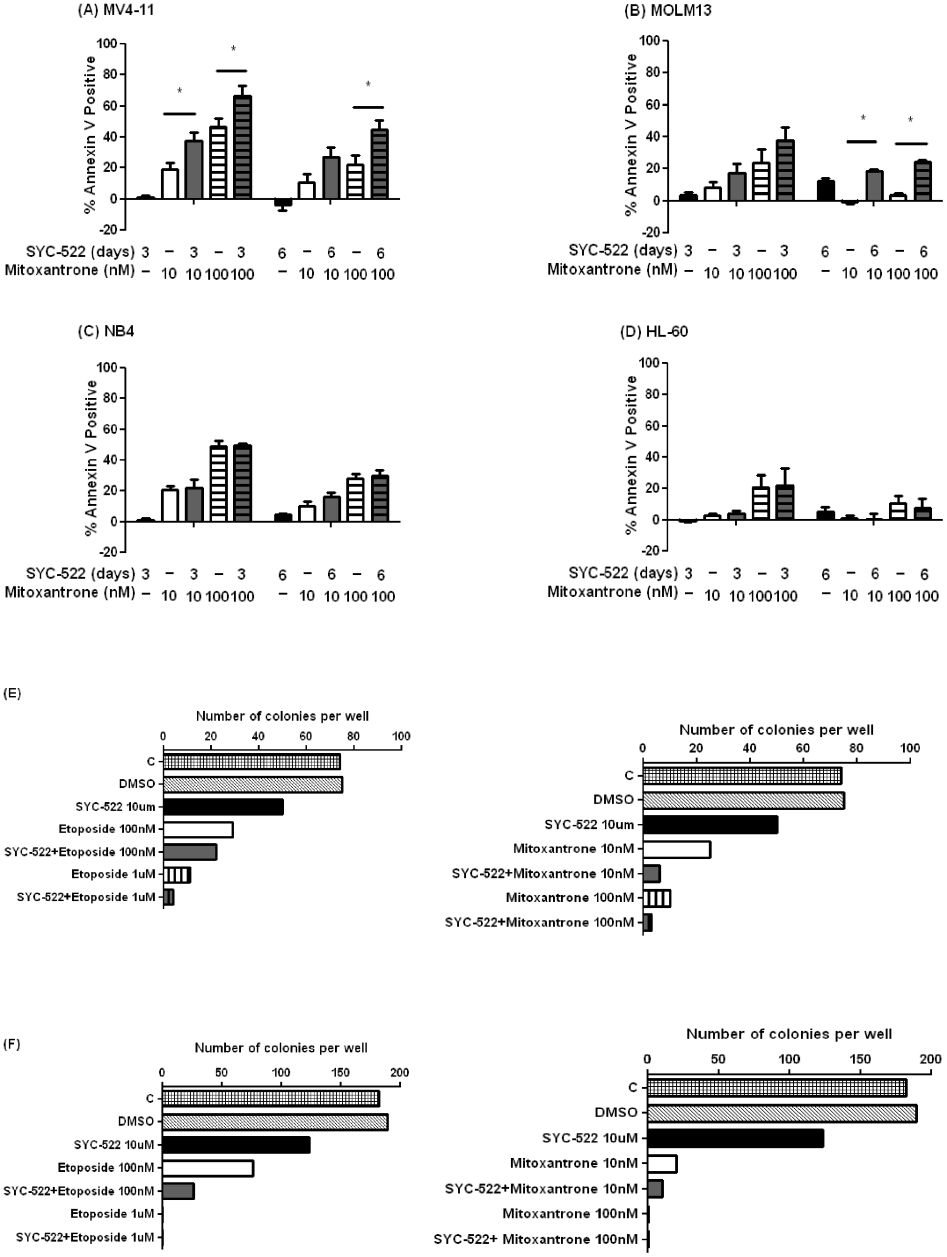
SYC-522 sensitized MLL-rearranged cells to mitoxantrone-induced apoptosis. (A) MV4-11 cells were treated with 3 µM SYC-522 and (B–D) other cell lines were treated with 10 µM SYC-522 for 0, 3, or 6 days. Following pretreatment, mitoxantrone (10 nM or 100 nM) was added to cells for 24 h incubation before the measurement of cell apoptosis by Annexin V staining. Bars represent the mean ±SEM of 3 independent experiments. *: p<0.05, chemotherapy vs. chemotherapy +SYC-522, unpaired t-test. (E–F) Two primary MLL-rearranged AML samples were cultured in methylcellulose and treated with SYC-522 (10 µM) and chemotherapy (etoposide 100 nM or 1 µM; or mitoxantrone 10 nM or 100 nM). After two weeks, the total numbers of colonies were counted for each sample, and the values from duplicate dishes were averaged.

Various proteins involved in DNA damage signaling have been associated with mechanisms of chemoresistance in cancer [15], [16]. Since DOT1L methyltransferase activity is also involved in the DNA damage response [14], we hypothesized that treating MLL-rearranged leukemia cells with SYC-522 would sensitize the cells to chemotherapy. To test this hypothesis, we treated the MV4-11 and MOLM13 cells with SYC-522 in combination with mitoxantrone (Fig. 5), etoposide (Fig. S2) and cytarabine (Fig. S3), three chemotherapeutic drugs commonly used to treat AML.

The results showed that SYC-522 alone did not induce cell apoptosis. In most cases, the SYC-522-induced apoptosis rate was less than 10% after treating the cells for up to 6 days (Fig. S4). However, SYC-522 sensitized the cells to apoptosis induced by traditional chemotherapeutic drugs. The cells were pretreated with SYC-522 for 3 or 6 days, followed by exposure to mitoxantrone, etoposide or cytarabine, together with SYC-522, for 24 h. These chemotherapy agents represent those most commonly used to treat pediatric patients with AML. The apoptosis rate was measured by Annexin V-FITC staining. On day 3, the apoptosis rate in MV4-11 cells treated with SYC-522 in combination with mitoxantrone was significantly higher than that in cells treated with mitoxantrone alone (Fig. 5A). Similar results were seen at Day 6 in MV4-11 and MOLM13 cells (Fig. 5A and 5B). As with mitoxantrone, pre-treatment of MV4-11 and MOLM13 cells with SYC-522 significantly increased etoposide and cytarabine-induced apoptosis (Fig. S2 and S3). Since the low SYC-522-induced apoptosis rate precluded testing for synergistic interactions, we calculated the potentiation factor for SYC-522+ mitoxantrone v. mitoxantrone alone (EC_50_ for mitoxantrone/EC_50_ for combination). For MV4-11, the potentiation factor was 6.5, indicating that SYC-522 significantly augmented the potency of mitoxantrone in MLL-rearranged leukemia cells. For NB4 and HL-60 without an MLL rearrangement, no differences in the chemotherapy-induced apoptosis rate were observed with and without SYC-522 pretreatment (Fig. 5C and 5D; Fig. S2 and S3).

We also treated two independent MLL-rearranged AML primary cell samples with SYC-522 and chemotherapeutic drugs. Cells were cultured in methylcellulose with SYC-522, chemotherapy, or both, for 14 days. For both AML samples, there were fewer colonies after combination treatment, as compared to those treated with mitoxantrone, etoposide, or SYC-522 alone (Fig. 5E and 5F). Taken together, our results showed that the sensitization to chemotherapy induced by SYC-522 was specific to MLL-rearranged leukemia cells, suggesting this disease could be effectively treated by the drug combination. In addition, our findings supported the conclusion that DOT1L is important for drug resistance of MLL-rearranged leukemia.

### SYC-522 treatment inhibited DNA damage repair and promoted mitoxantrone-induced cell apoptosis

**Figure 6:**
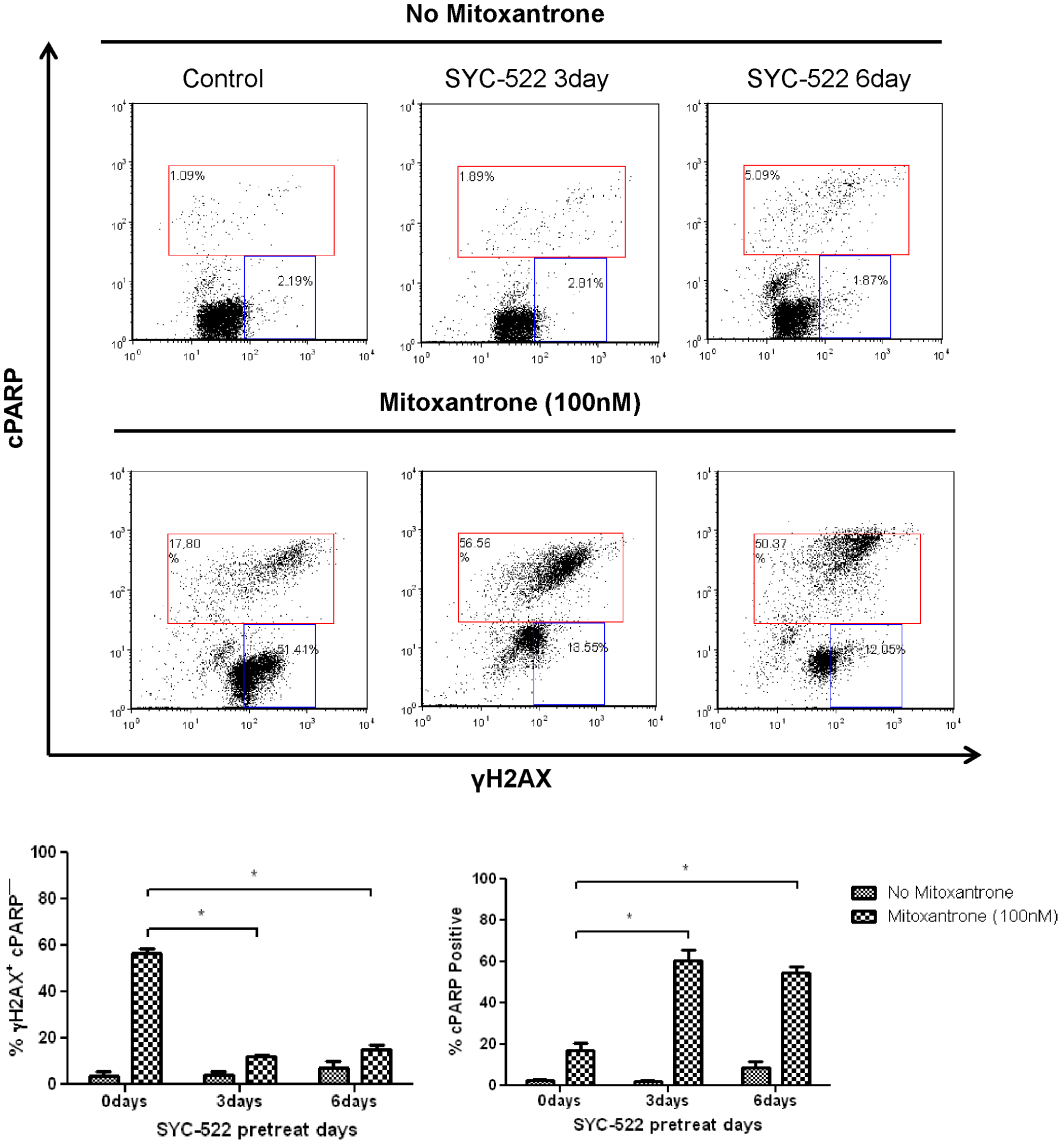
SYC-522 impaired DNA damage response and promoted mitoxantrone- induced apoptosis in MV4-11 cells. MV4-11 cells were pretreated with SYC-522 (3 µM) for 3 or 6 days, followed by 100 nM mitoxantrone for 4 h. Then mitoxantrone was washed away and cells were incubated in fresh medium for 12 h. The cells were then analyzed by flow cytometry for the percent viable and repairing (γH2AX+/cPARP−; blue gate) and the percent undergoing apoptosis (cPARP+; red gate). A representative series of dot plots is shown. Bars represent the mean ±SEM for 3 independent experiments. *: p<0.05, chemotherapy vs. chemotherapy +SYC-522, unpaired t-test.

Since H3K79 methylation is reported to be involved in the DNA damage response [14], we reasoned that SYC-522 might impair chemotherapy-induced DNA damage signaling. The phosphorylated histone H2AX (γH2AX) recruits DNA damage repair enzymes, and it can be used as a marker of active DNA damage signaling in translational cancer research [25]. After treating SYC-522-pretreated MV4-11 cells with mitoxantrone (a topoisomerase II inhibitor) for 4 h, the cells were incubated for 12 h in fresh medium to allow DNA damage signaling and repair. After the recovery period, we analyzed the level of γH2AX and cleaved PARP (cPARP), which was used as a marker for apoptosis, by flow cytometry. Cells treated with only mitoxantrone showed more than 40% higher level of γH2AX+/cPARP− cells, compared with cells pretreated with SYC-522 for 3 or 6 days prior to mitoxantrone. Furthermore, after exposure to mitoxantrone for 4 h, SYC-522-pretreated cells had a significantly higher percent of cPARP+ cells than non-pretreated cells (Fig. 6). Similar results were seen in MOLM13 cells (Fig. S5). The increase in cPARP+ cells and decrease in viable γH2AX+ cells suggest that SYC-522 pretreatment may impair effective DNA damage repair signaling and promote cells to undergo apoptosis.

## Discussion

SYC-522 is a potent inhibitor of DOT1L and represents a useful probe for studying the biological functions of DOT1L in MLL-rearranged leukemia. Our results demonstrated that DOT1L inhibition significantly and specifically reduced H3K79 methylation, and thereby effectively blocked the expression of *HOXA9* and *MEIS1*, which are upregulated by H3K79me2. It has been reported that H3K4 methylation is required for the maintenance of HOX family gene expression during hematopoietic development [21]. Our compound did not significantly alter H3K4me3 in MLL-rearranged cells (Fig. 1). H3K27 methylation is involved in the repression of developmental and differentiation genes [22], [26]. Though we observed increased methylation of H3K27 in MV4-11 cells on day 3 (Fig. 1A), this mark remained unchanged in MOLM13 cells (Fig. 1B). Further, SYC-522 treatment caused MV4-11 cell differentiation (Fig. 4A), which is the opposite result expected from increased H3K27me3, suggesting that the primary effects of this drug are not due to alterations at H3K27. Our results indicate that SYC-522 inhibited DOT1L activity, resulting in downregulation of key leukemogenic genes.

The decrease in H3K79me2 also significantly reduced the expression of *CCND1* (cyclin D1). The suppression of cyclin D1 results in cell cycle arrest at G0/G1 phase [27]. After 6-day SYC-522 treatment, expression was decreased more than 90% in MV4-11 cells (Fig. 2A). Consistent with this finding, SYC-522 treatment of MV4-11 cells resulted in cell cycle arrest at the G0/G1 phase (Fig. S1).

*BCL2L1* (BCL-xL) prevents the release of cytochrome c and other proapoptotic molecules from mitochondria, and therefore inhibits apoptosis [28], [29]. SYC-522 treatment also decreased the expression of *BCL2L1* in both MLL-rearranged leukemia cell lines (Fig. 2A and 2B). Interestingly, we observed very low apoptosis rates after treating the MLL-rearranged leukemia cells with SYC-522 for prolonged periods (Figs. 5 and 6). These results suggested that decreasing expression of this anti-apoptosis gene is not sufficient to induce apoptosis. It is important to note that both *CCND1* and *BCL2L1* gene expression also were decreased significantly in leukemia cell lines NB4 and HL-60, which do not have MLL rearrangement (Fig. 2C and 2D). Since H3K79 methylation has now been recognized to be a biomarker of active transcription [30], [31], it is not surprising that inhibition of H3K79 methylation might affect a broad range of gene expression. It is not known whether *CCND1* and *BCL2L1* are regulated by H3K79 methylation, so it is also possible that the downregulation of these genes was due to off-target or indirect effects of SYC-522 treatment. Previous studies show another DOT1L inhibitor, EPZ004777, also inhibits the proliferation of leukemia cells without an MLL-rearrangement, although to a lesser degree as compared to MLL-rearranged cells [18]. However, the requirement for H3K79-methylation-dependent gene overexpression in MLL-rearranged leukemia rendered these cells far more sensitive to DOT1L inhibitors.

In contrast to other reported DOT1L inhibitors [17], [18], our drug did not induce apoptosis as a single agent. Instead, SYC-522 may decrease cell growth by inducing differentiation of MLL-rearranged leukemia cells. As shown in Figure 4, SYC-522 induced monocytic differentiation in MLL-rearranged leukemia cell lines and primary MLL-rearranged AML samples. SYC-522 treatment also inhibited the colony formation ability of primary MLL-rearranged AML samples. The primary ALL samples we studied were somewhat less sensitive to this effect of the drug in the colony formation assay. Importantly, SYC-522 had very little effect on normal bone marrow CFUs. To our knowledge, this is the first report of DOT1L inhibition in primary human leukemia and normal human bone marrow cells. Our results suggest that the effects of SYC-522 may be somewhat specific for MLL-rearranged AML.

Strikingly, treating MLL-rearranged leukemia cells with SYC-522 before applying chemotherapy drugs significantly increased their apoptosis (Fig. 5, Fig. S2 and S3). This was a novel and important finding as DOT1L inhibitors move into clinical trials. It has been reported that DOT1L can promote DNA damage signaling and repair [14]. DOT1L not only methylates H3K79, thereby opening chromatin for repair, but it also recruits and activates 53BP1, a conserved DNA double strand break sensor. Enhanced DSB repair is an effective mechanism of chemoresistance [16], [32], [33]. Therefore, it is possible that inhibition of DOT1L by SYC-522 sensitized MLL-rearranged leukemia cells to chemotherapy by disrupting DNA damage signaling and preventing DSB repair. One of the earliest events in the DNA damage response is phosphorylation of histone H2AX (γH2AX) by ATM, ATR or DNA-PK [34], [35], [36]. Increased γH2AX surrounding a DSB site forms a focus of open chromatin and serves as a platform to recruit other molecules involved in DNA damage repair [37]. Poly (ADP-ribose) polymerase (PARP) is also involved in DNA damage repair [38], [39], but the cleavage of PARP by caspase-3 inhibits this activity and instead promotes cell apoptosis [40]. Our DNA damage recovery assay showed that pretreating MLL-rearranged leukemia cells with SYC-522 significantly reduced the proportion of viable γH2AX+ cells, and increased the apoptotic (cPARP+) cells after mitoxantrone treatment. Therefore, it is possible that SYC-522 pretreatment impaired effective DNA damage signaling and repair, and thus sensitized MLL-rearranged leukemia cells to chemotherapy. Further experiments are underway to test this hypothesis.

One challenge that remains to be overcome before this compound can be adequately studied in vivo is its poor aqueous solubility and rapid metabolism by liver microsomes [41]. The Epizyme compounds also required prolonged administration strategies when used in vivo [17], [18]. Our current focus is on optimizing these pharmacokinetic properties to enable studies of the inhibitor in combination with chemotherapy in the murine xenograft model.

In summary, our results suggested that DOT1L inhibition may be a potent strategy for the treatment of MLL-rearranged leukemia by inhibiting DOT1L methyltransferase activity and its downstream targets, thereby promoting differentiation. Further, DOT1L inhibitors may be combined with other DNA-damaging anticancer drugs to reverse chemoresistance of MLL-rearranged leukemia cells.

## Supporting Information

Figure S1 **SYC-522 induced cell cycle arrest in MLL-rearranged cells.** MV4-11 cells were treated with 3 µM SYC-522 for up to 9 days and analyzed by flow cytometry (FACScan) every 3 days for cell cycle with PI staining. Bars represent the mean ±SEM of 3 independent experiments. *: p<0.05, day 0 vs. day 3, 6, or 9, unpaired t-test.
(TIF)

Figure S2 **SYC-522 sensitized MLL-rearranged cells to etoposide.** (A) MV4-11 cells were treated with 3 µM SYC-522 and (B–D) other cell lines were treated with 10 µM SYC-522 for 0, 3, or 6 days. Following pretreatment, etoposide (100 nM or 1 µM) was added to cells for 24 h incubation before the measurement of cell apoptosis by Annexin V staining. Bars represent the mean ±SEM of 3 independent experiments. *: p<0.05, chemotherapy vs. chemotherapy +SYC-522, unpaired t-test.
(TIF)

Figure S3 **SYC-522 sensitized MLL-rearranged cells to cytarabine.** (A) MV4-11 cells were treated with 3 µM SYC-522 and (B–D) other cell lines were treated with 10 µM SYC-522 for 0, 3, or 6 days. Following pretreatment, cytarabine (3 µM or 30 µM) was added to cells for 24 h incubation before the measurement of cell apoptosis by Annexin V staining. Bars represent the mean ±SEM of 3 independent experiments. *: p<0.05, chemotherapy vs. chemotherapy +SYC-522, unpaired t-test.
(TIF)

Figure S4 **SYC-522 treatment did not induce apoptosis in MV4-11 cells.** MV4-11 cells were plated in a 24-well plate and treated with 3 µM SYC-522. Every six days, 80% cells were removed and fresh medium and SYC-522 were added. The apoptosis rates were measured at day 1, 3, 6, 9, 15, and 20. Values represent the mean ±SEM for 3 independent experiments.
(TIF)

Figure S5 **SYC-522 treatment inhibited γH2AX activation and promoted cell apoptosis.** MOLM13 cells were pretreated with SYC-522 (10 µM) for 3 or 6 days, followed by 100 nM mitoxantrone for 4 h. Then mitoxantrone was washed away and cells were incubated in fresh medium for 12 h. The cells were then analyzed by flow cytometry for the percent viable and repairing (γH2AX+/cPARP−) and the percent undergoing apoptosis (cPARP+). A representative series of dot plots is shown. Bars represent the mean ±SEM for 3 independent experiments. *: p<0.05, chemotherapy vs. chemotherapy +SYC-522, unpaired t-test.
(TIF)
