# Supplementary figures and images for: DOT1L Inhibition Sensitizes MLL-Rearranged AML to Chemotherapy
Source: PLoS One. 2014 May 23;9(5):e98270. doi: 10.1371/journal.pone.0098270 (PMC4032273; doi:10.1371/journal.pone.0098270)

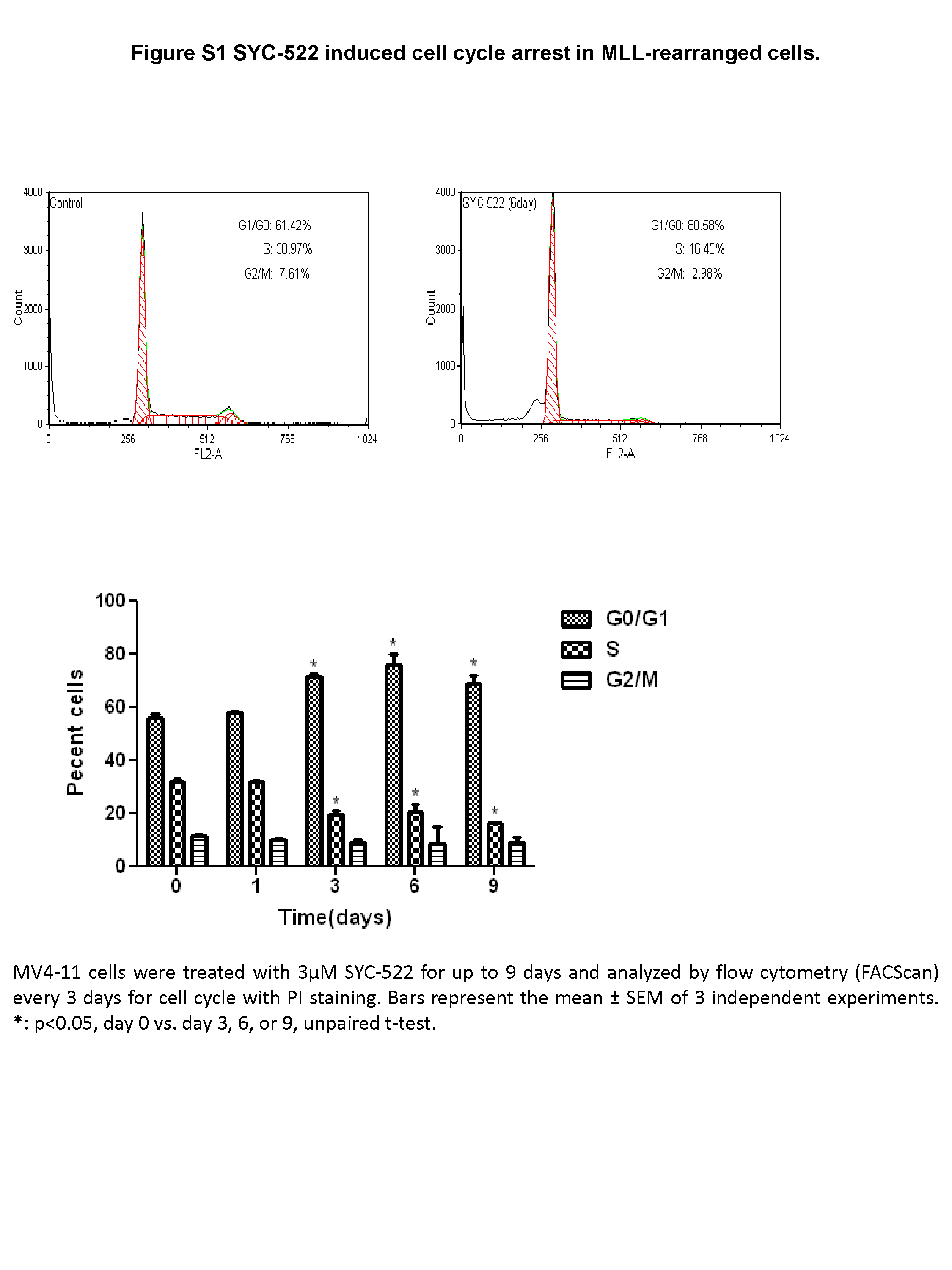

Supplement: Figure S1 — SYC-522 induced cell cycle arrest in MLL-rearranged cells. MV4-11 cells were treated with 3 µM SYC-522 for up to 9 days and analyzed by flow cytometry (FACScan) every 3 days for cell cycle with PI staining. Bars represent the mean ±SEM of 3 independent experiments. *: p<0.05, day 0 vs. day 3, 6, or 9, unpaired t-test. (TIF) [file pone.0098270.s001.tif]

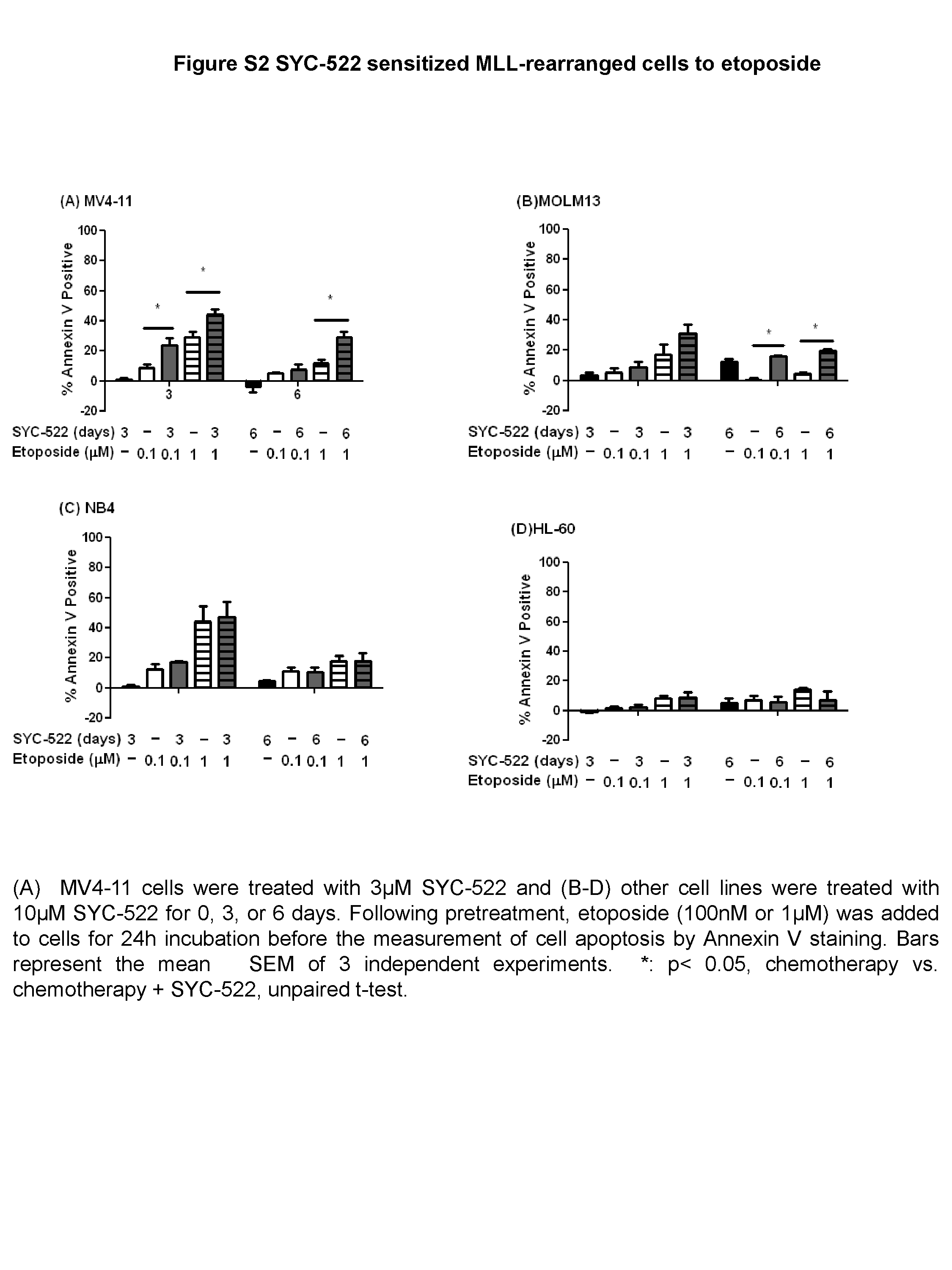

Supplement: Figure S2 — SYC-522 sensitized MLL-rearranged cells to etoposide. (A) MV4-11 cells were treated with 3 µM SYC-522 and (B–D) other cell lines were treated with 10 µM SYC-522 for 0, 3, or 6 days. Following pretreatment, etoposide (100 nM or 1 µM) was added to cells for 24 h incubation before the measurement of cell apoptosis by Annexin V staining. Bars represent the mean ±SEM of 3 independent experiments. *: p<0.05, chemotherapy vs. chemotherapy +SYC-522, unpaired t-test. (TIF) [file pone.0098270.s002.tif]

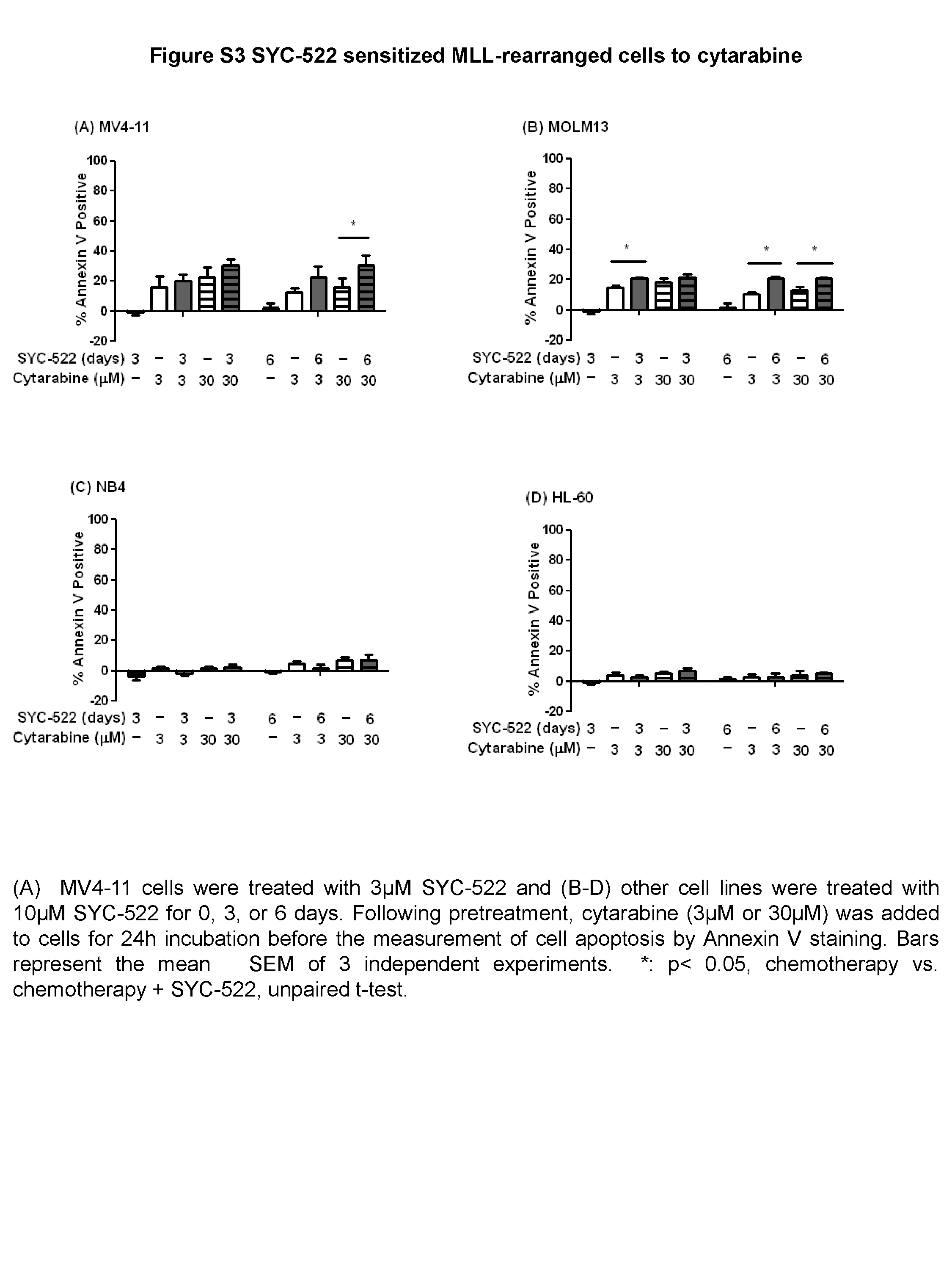

Supplement: Figure S3 — SYC-522 sensitized MLL-rearranged cells to cytarabine. (A) MV4-11 cells were treated with 3 µM SYC-522 and (B–D) other cell lines were treated with 10 µM SYC-522 for 0, 3, or 6 days. Following pretreatment, cytarabine (3 µM or 30 µM) was added to cells for 24 h incubation before the measurement of cell apoptosis by Annexin V staining. Bars represent the mean ±SEM of 3 independent experiments. *: p<0.05, chemotherapy vs. chemotherapy +SYC-522, unpaired t-test. (TIF) [file pone.0098270.s003.tif]

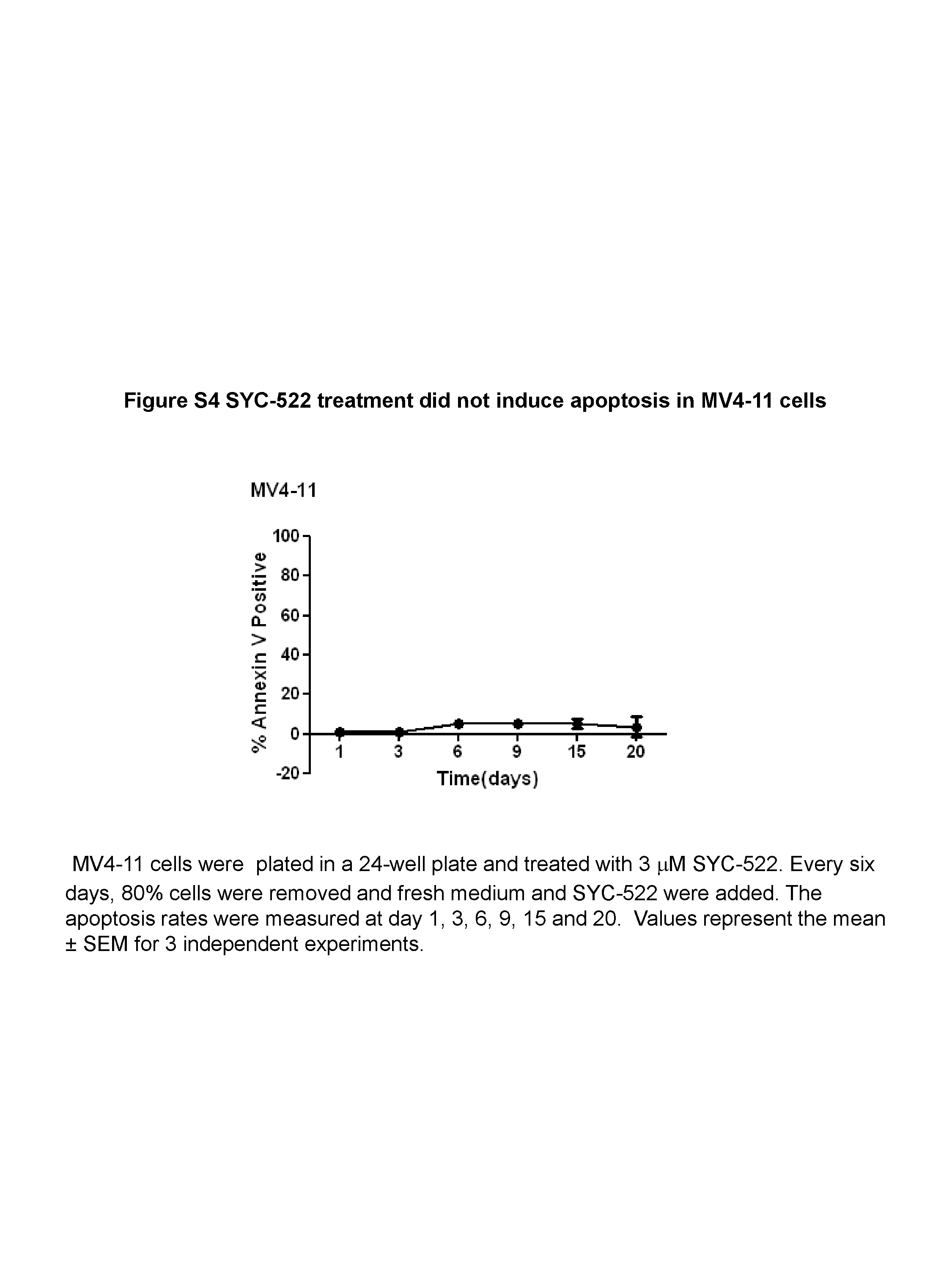

Supplement: Figure S4 — SYC-522 treatment did not induce apoptosis in MV4-11 cells. MV4-11 cells were plated in a 24-well plate and treated with 3 µM SYC-522. Every six days, 80% cells were removed and fresh medium and SYC-522 were added. The apoptosis rates were measured at day 1, 3, 6, 9, 15, and 20. Values represent the mean ±SEM for 3 independent experiments. (TIF) [file pone.0098270.s004.tif]

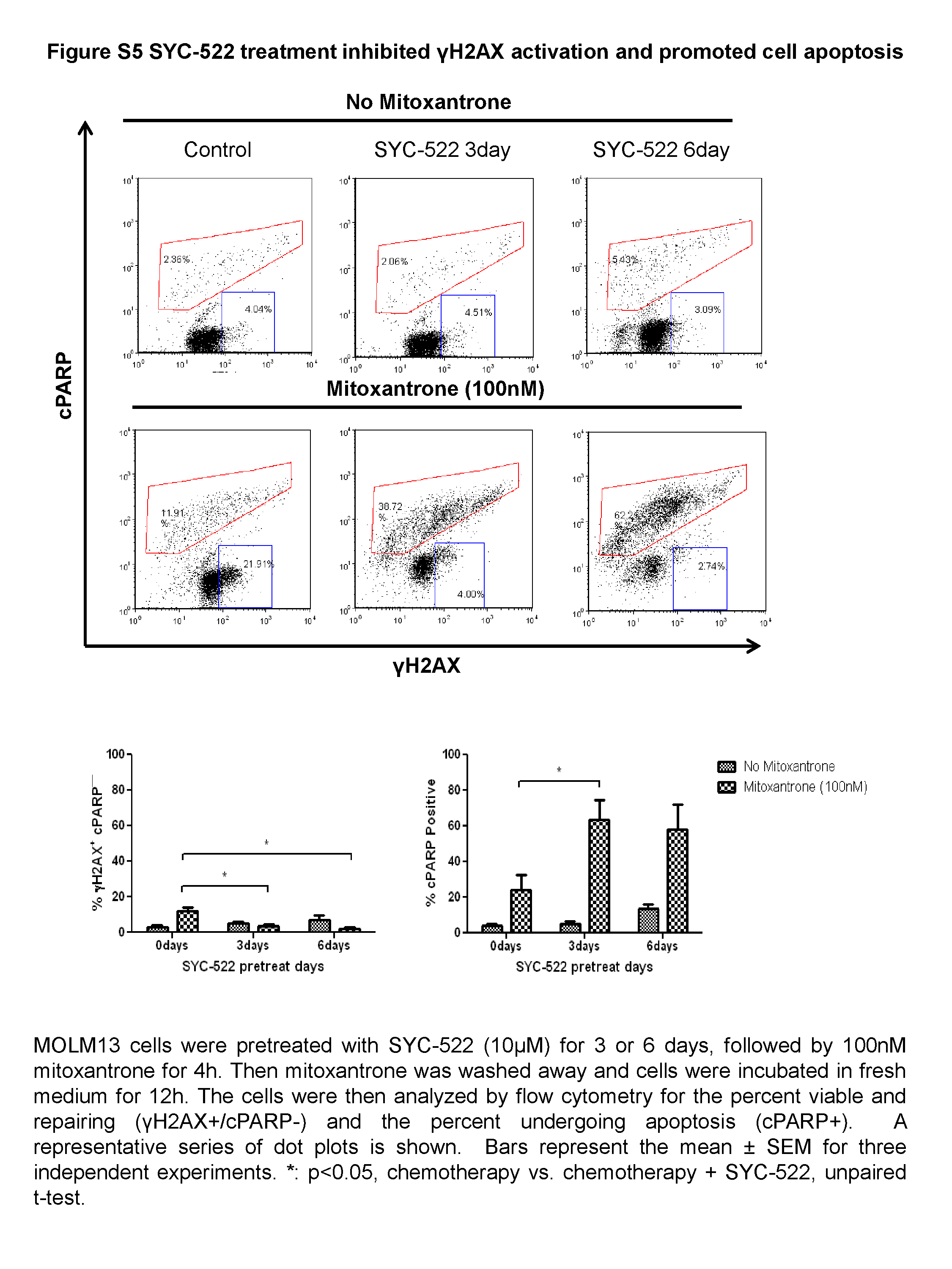

Supplement: Figure S5 — SYC-522 treatment inhibited γH2AX activation and promoted cell apoptosis. MOLM13 cells were pretreated with SYC-522 (10 µM) for 3 or 6 days, followed by 100 nM mitoxantrone for 4 h. Then mitoxantrone was washed away and cells were incubated in fresh medium for 12 h. The cells were then analyzed by flow cytometry for the percent viable and repairing (γH2AX+/cPARP−) and the percent undergoing apoptosis (cPARP+). A representative series of dot plots is shown. Bars represent the mean ±SEM for 3 independent experiments. *: p<0.05, chemotherapy vs. chemotherapy +SYC-522, unpaired t-test. (TIF) [file pone.0098270.s005.tif]
